# Evaluating the Clinical Utility of the Interpersonal Hopelessness Scale Among Adults With Past-Year Suicide Ideation

**DOI:** 10.1111/sltb.70108

**Published:** 2026-06

**Authors:** Michael LeDuc, Cole Marvin, Sean M. Mitchell, Sarah L. Brown, Emma H. Moscardini, Raymond Tucker

**Affiliations:** 1 Department of Psychological Sciences, Texas Tech University, Lubbock, Texas, USA; 2 Department of Psychology, Florida State University, Tallahassee, Florida, USA; 3 Department of Psychology, Louisiana State University, Baton Rouge, Louisiana, USA

**Keywords:** clinical assessment, interpersonal hopelessness, interpersonal theory of suicide, perceived burdensomeness, suicide ideation, suicide risk, thwarted belongingness

## Abstract

**Introduction:**

The Interpersonal Hopelessness Scale (IHS) measures hopelessness about thwarted belongingness (TB) and perceived burdensomeness (PB) improving over time, which represent core components of the interpersonal theory of suicide. We aimed to evaluate IHS clinical utility by examining whether IHS total and subscale scores predicted both concurrent and short-term prospective suicidal desire.

**Methods:**

US adults with past-year suicide ideation completed online self-report assessments at two time points, 1 week apart (Wave 1 [W1], *n* = 595; Wave 2 [W2], *n* = 200).

**Results:**

IHS total, TB, and PB scores were each significantly associated with the presence of concurrent suicidal desire and adequately discriminated between those with and without suicidal desire. Prospectively, all W1 IHS scores predicted W2 suicidal desire; however, area under the curve statistic values were lower than in concurrent models. When including both IHS subscales as simultaneous predictors, only IHS-PB remained a significant predictor of prospective suicidal desire. We present clinical cutoff scores and estimated probabilities of suicidal desire to support IHS score interpretation in applied settings.

**Conclusion:**

These findings provide preliminary support for the IHS as a theory-informed and clinically meaningful tool for suicide risk assessment, with the potential to enhance case conceptualization and guide intervention planning.

Suicide rates in the United States (US) have continued to rise, reaching an all-time peak of over 49,000 deaths by suicide in 2022 (Centers for Disease Control and Prevention [CDC] 2024). Perhaps more alarming is the substantially greater number of individuals who experienced serious suicidal thoughts (13.2 million), made a suicide plan (3.8 million), or attempted suicide (1.6 million) in the past year (CDC, 2024). Although the vast majority of US adults who think about suicide do not go on to attempt or die by suicide, suicidal thoughts are clearly a significant public health concern—one that warrants continued empirical and clinical attention to alleviate suffering and prevent suicidal crises (Jobes and Joiner 2019). To better understand and address the full spectrum of suicide risk, contemporary ideation-to-action framework theories of suicide distinguish mechanisms involved in the development and maintenance of suicide ideation (SI) from processes that facilitate the transition from SI to lethal or near-lethal suicidal behaviors. The interpersonal theory of suicide (ITS; Joiner 2005; Van Orden et al. 2010) is one of the most studied and empirically supported ideation-to-action framework theories of suicide (Chu et al. 2017). However, measures used to assess the ITS have received relatively little attention regarding their application in clinical settings to inform risk formulation and treatment. Thus, we aimed to examine the clinical utility of the recently developed Interpersonal Hopelessness Scale (IHS; Mitchell et al. 2024; Tucker et al. 2018) to enhance our understanding of the ITS and SI in both research and clinical settings.

The ITS proposes that experiencing either thwarted belongingness (TB; indicated by loneliness and a lack of reciprocal caring relationships) *or* perceived burdensomeness (PB; indicated by self-hate and feelings of liability on others) can lead to passive SI (i.e., thoughts of death, but not suicide; Van Orden et al. 2010). However, when an individual experience both a sense of TB *and* PB *and* feels hopeless about these interpersonal states changing, active SI (i.e., active thoughts about ending one’s life, desire to die by suicide) is posited to develop. According to the ITS, active SI is considered a necessary condition for the escalation of suicidal crises; within this context, suicide capability (e.g., a decreased fear of death, increased pain tolerance) facilitates the development of suicidal intent and progression toward a lethal or near-lethal suicide attempt. Thus, accurately identifying and assessing active SI is critical for both empirical investigation and clinical intervention.

Although the ITS hypotheses have largely been supported (Chu et al. 2017; Ma et al. 2016), TB and PB have been assessed most commonly using the 15-item version of the Interpersonal Needs Questionnaire (INQ-15; Chu et al. 2017; Van Orden et al. 2012), which does not capture *interpersonal hopelessness* (i.e., hopelessness about TB and PB). The lack of assessment of interpersonal hopelessness has precluded direct tests of the theory’s central hypothesis related to the development of active SI. Indeed, this limitation has been noted in meta-analytical findings spanning over 10 years of ITS research (Chu et al. 2017) and in a recent commentary on the ITS’s current evidence base and priorities for future research (Joiner et al. 2021). Recently, interpersonal hopelessness has gained increased empirical attention, prompting the thorough development and evaluation of the Interpersonal Hopelessness Scale (IHS; e.g., Mitchell et al. 2024; Tucker et al. 2018). The IHS is a 10-item self-report assessment of hopelessness specific to TB (IH-TB; 5 items) and PB (IH-PB; 5 items). Research has supported both one- and two-factor solutions using exploratory and confirmatory factor analyses (Mandracchia et al. 2021; Mitchell et al. 2024; Tucker et al. 2018); however, most recently, Mitchell et al. (2024) demonstrated that the two-factor model fit the data significantly better than the one-factor model. Research has further demonstrated that IH-TB and IH-PB significantly predict active SI concurrently and prospectively and are strongly associated with TB, PB, and general hopelessness without being statistically equivalent to them (*r*s = 0.39–0.79; Mitchell et al. 2024). Thus, the IHS may offer a more theory-consistent option to assess hopelessness about TB and PB when active SI is the outcome of interest. Additionally, recent ambulatory assessments of interpersonal hopelessness have also indicated that IH-TB and IH-PB have greater temporal stability than general hopelessness and were concurrent and prospective predictors of suicidal desire (Gerner et al. 2023).

These findings strongly support the broader adoption of the IHS for theory testing and highlight its potential value for clinical risk assessment and intervention efforts. Importantly, the IHS, like the INQ-15, is brief, theoretically grounded, and may be less susceptible to underreporting of SI than more face-valid assessments of suicide risk that specifically ask about current or historical SI, suicide attempts, and/or non-suicidal self-injury, making it particularly well-suited for clinical settings. Notably, examinations of the clinical utility of the INQ-15 (e.g., Mitchell et al. 2017, 2020; Silva et al. 2023) occurred at least 5 years after the publication of Van Orden et al.’s (2012) seminal paper on the INQ-15, which slowed the measure’s dissemination to clinical practice and reinforced a gap between research and clinical implementation. Given the potential advantages of the IHS in clinical settings and the need to promptly advance translational research in this domain, further evaluation is necessary to assess the clinical utility of the IHS.

To accelerate the application of the IHS in clinical practice, we aimed to examine its clinical utility. Specifically, we tested the IHS total and subscale scores as predictors of concurrent (Wave 1 [W1]) and prospective (1 week later; Wave 2 [W2]) suicidal desire. To address potential issues that arise due to multicollinearity and the effects of partialling out clinically relevant variance that can mask clinical utility (Lynam et al. 2006; Mitchell et al. 2017), we examined the IHS subscales and total scores in separate and multiple predictor models and compared the relevant area under the curve (AUC) statistics as a measure of effect size. Consistent with prior research on the clinical utility of the INQ and reporting recommendations (Mitchell et al. 2017, 2020, 2021), we provided the estimated probabilities of the IHS scores and clinical cutoff scores that maximize sensitivity and specificity derived from model receiver operating characteristic (ROC) curves. This information provides clinicians with easily interpretable tools to further characterize a patient’s level of risk based on their IHS scores, which can guide clinical decision-making.

## 1 | Method

### 1.1 | Participants

Participants were adults in the United States recruited through the Qualtrics Panel System and who reported a non-zero frequency of past-year SI on the Suicidal Behaviors Questionnaire-Revised item two (Osman et al. 2001; see Procedures for additional details). At W1, there were 595 adult participants aged 18–83 years (M = 35.75 years, SD = 14.69) with complete data on all W1 variables of interest. At W2, there were 215 adult participants aged 18–71 years (M = 36.50 years, SD = 14.41) with complete data on the W2 criterion variable, and 200 participants aged 18–71 years (M = 37.11 years, SD = 14.57) with complete data on all W2 variables of interest. Refer to Table 1 for additional demographic characteristics of the W1 sample, W2 sample, and final analytic sample.

### 1.2 | Measures

#### 1.2.1 | Demographic and History Form

Participants responded to demographic questions assessing sex, age, gender, sexual orientation, race, ethnicity, highest degree earned, and past-year household income.

#### 1.2.2 | Attention Checks

We identified potential participants with random responding patterns or inattention at each wave with three attention check items embedded in the self-report assessments throughout the survey. For example, participants were asked to “select ‘strongly agree’ if you are paying attention” (Alvarez et al. 2019). We excluded participants who missed more than one attention check, which we further describe below.

#### 1.2.3 | Interpersonal Hopelessness Scale (IHS)

The IHS (Tucker et al. 2018) is a 10-item self-report measure of an individual’s interpersonal hopelessness, or hopelessness about TB (5 items) and PB (5 items), as described within the ITS. The scale assesses hopelessness about these interpersonal states persisting over time. For example, IH-TB items assess hopelessness about feeling connected to others (e.g., “I expect that people will never care about me.”) whereas IH-PB items assess hopelessness about no longer feeling like a burden to others (e.g., “I will always fail the people in my life.”). Participants select response options on a scale ranging from 0 (*Definitely false*) to 5 (*Completely true*). Although initial exploratory factor analyses supported a one-factor structure (Mandracchia et al. 2021; Tucker et al. 2018), confirmatory factor analyses demonstrated that the two-factor structure (IH-TB and IH-PB) fit the data better than a one-factor structure; however, both the one- and two-factor structures demonstrated good data fit (Forkmann et al. 2022; Mitchell et al. 2024). In the current study, the W1 IH-TB (α = 0.92), IH-PB (α = 0.92), and IH-Total demonstrated (α = 0.94) excellent internal consistency reliability.

#### 1.2.4 | Beck Scale for Suicide Ideation, Item 4 (BSS)

The BSS (Beck and Steer 1991) is a 21-item self-report measure of SI, intent, plans, and preparatory behaviors during the past week (19 items), as well as the incidence and medical severity of the individual’s lifetime suicide attempt history (2 items). The BSS has demonstrated excellent psychometric properties (Beck and Steer 1991; Brown et al. 2000). In the current study, the BSS yielded excellent internal consistency reliability at W1 (α = 0.94) and W2 (α = 0.95). We assessed *suicidal desire* using BSS item 4, which asks individuals to select the degree of their desire for suicide during the past week using a three-point ordinal response scale (0 = no desire for death/suicide; 1 = weak desire for death/suicide; 2 = moderate to strong desire for death/suicide). Suicidal desire is consistent with the ITS description of active SI (Van Orden et al. 2010) and a particularly important facet of SI (compared to intent or controllability of a suicidal urge) when predicting a future suicide attempt (Wang et al. 2021). We dichotomized BSS item 4 into no desire for suicide (coded 0) or a weak, moderate, or strong desire for suicide (coded 1). See Data Analyses below for further detail about and justification for dichotomization. Point-biserial and Pearson correlations between study variables are presented in Table 2. In the current study, at W1 (*N* = 595), 350 (58.8%) of participants reported some level of suicidal desire; at W2 (*N* = 200), 89 (44.5%) reported some level of suicidal desire.

### 1.3 | Procedures

The overseeing university institutional review board approved all procedures for this study. Participants were recruited using the Qualtrics Panel System, an online recruitment platform with a database that includes millions of US residents. Using this system, we recruited US residents who were at least 18 years old and who reported a non-zero frequency of past-year SI on the Suicidal Behaviors Questionnaire-Revised item two (Osman et al. 2001). We selected this recruitment approach to obtain a diverse adult sample with recent SI for an initial evaluation of the clinical utility of the IHS in a higher-risk community sample. Eligible individuals were emailed an invitation containing study links. After providing online informed consent, participants completed the measures online.

Data for the current study were drawn from a larger two-wave longitudinal study (Moscardini and Tucker 2023). At W1, participants completed a suicide Stroop Task (data not included in the current study), followed by the demographic and history form, and then the other self-report measures. After 1 week, W1 participants were re-contacted once by email through Qualtrics Panels and invited to complete W2. At W2, participants again completed the suicide Stroop Task (data not included in the current study) and a shorter self-report battery (to reduce attrition). At the conclusion of the study, all participants were presented with a debriefing form and national mental health and suicide resources (e.g., suicide crisis line numbers). All participants were compensated via the Qualtrics Panel system for participating in both waves at rates pre-determined by Qualtrics Panels. The average time to complete W1 was 24.46 min (SD = 9.58) and W2 was 9.91 min (SD = 5.42). This study was not pre-registered, and no publicly available materials are available for this study; however, all materials can be provided upon request from the corresponding author. We report all data exclusions, all manipulations, and all measures in the study.

### 1.4 | Data Analyses

We conducted analyses using SAS 9.4 statistical software. We used raw scores for IH-TB, IH-PB, and IH-Total in the analyses, as these are easier to calculate (compared to standardized scores) in clinical settings. To better compare effect sizes, we also standardized (z-score transformed) the predictor variables to provide standardized odds ratios (*SOR*) and confidence intervals, as recommended by Mitchell et al. (2021). The BSS total score lacks an established clinical cutoff score (Beck and Steer 1991); therefore, there was no cutoff to dichotomize the continuous BSS scores. Given that the clinical utility analyses used in the current study require a binary criterion variable, we dichotomized BSS item 4 (no desire for suicide, coded 0; weak or greater desire, coded 1). This is consistent with other clinical utility work related to the ITS (Mitchell et al. 2017, 2021) and is necessary for analyses to examine the sensitivity, specificity, and clinical cutoff scores. Additionally, although this single item does not fully capture the suicidal desire construct, a point-biserial correlation analysis indicated the dichotomized BSS item 4 (*r*_pb_ = 0.72, *p* < 0.001) was significantly associated with the BSS total score. There were no missing data values on the W1 variables included in the analyses, and no missing responses on the W2 BSS criterion variables for participants in W2.

We conducted binary logistic regression analyses. We examined W1 IH-TB, IH-PB, and IH-Total scores in bivariate and multivariate models predicting W1 and W2 suicidal desire. Both the subscale scores and total scores were investigated, as previous research has demonstrated evidence for both one- and two-factor solutions (Mitchell et al. 2024; Tucker et al. 2018). Additionally, because IH-TB and IH-PB are highly correlated, they were examined in separate models to reduce multicollinearity concerns and avoid obscuring the interpretation of their unique effects (Mitchell et al. 2024). From an applied perspective, both one- and two-factor solutions can be easily calculated in clinical practice and may be of interest to examine separately or together in clinical case conceptualization. Per recommendations, we also graphed the estimated probability of suicidal desire across the IH-TB, IH-PB, and IH-Total scores (Mitchell et al. 2021). Next, we used Youden’s J statistic, which equally minimizes false negative and false positive rates to identify the clinical cutoff scores for IH-TB, IH-PB, and IH-Total.

We then conducted ROC curves to further demonstrate the effect sizes of the models via AUC statistics. We also conducted ROC curve analyses for IH-TB, IH-PB, and IH-Total scores as separate predictors of suicidal desire at W1 and W2. ROC curve analyses yield AUC values calculated from ROC curve models that test our hypotheses. ROC curve analyses also yield odds ratios (ORs) for each predictor variable. Generally, AUC values of 0.50 indicate no discrimination, between 0.70 and 0.80 adequate discrimination, between 0.80 and 0.90 good discrimination, and ≥ 0.90 excellent discrimination (Hosmer et al. 2013). Finally, we used ROC contrast tests to examine whether AUC values calculated from logistic regression analyses, including IH-TB, IH-PB, and IH-Total scores, differed when predicting W1 (i.e., concurrent) versus W2 (i.e., prospective) suicidal desire.

## 2 | Results

### 2.1 | Data Screening and Preparation

Of the 660 participants at W1, 65 were excluded because they missed more than one attention check, resulting in 595 participants included in the W1 analyses and demographic statistics. At W2, 21 of the 236 participants were excluded for the same reason, leaving 215 participants included in W2 demographic statistics. Of those 215 participants, 200 had complete data on all W2 variables of interest and were therefore included in analyses involving both W1 and W2 data. Across data from both waves, 3.39% of the data were missing, and Little’s Missing Completely at Random (MCAR) test indicated that the data were MCAR (*χ*^2^ [23,093, *N* = 207] = 14867.95, *p* > 0.999; Tabachnick and Fidell 2013). Therefore, missing data points were addressed using the expectation–maximization algorithm. There were no univariate or multivariate outliers. Logistic regression makes no assumptions about the distribution of the predictors; therefore, skewness and kurtosis were not examined (Tabachnick and Fidell 2013). The linearity in the logit assumption was satisfied for all models.

### 2.2 | Concurrent Logistic Regression Analyses

First, W1 IH-TB, IH-PB, and IH-Total scores were examined as separate predictors of W1 suicidal desire. W1 IH-TB (SOR = 2.60, 95% Wald CI [2.13, 3.16]), W1 IH-PB (SOR = 2.76, 95% Wald CI [2.25, 3.39]), and W1 IH-Total (SOR = 2.92, 95% Wald CI [2.37, 3.59]) were significantly associated with W1 suicidal desire. The AUC values for W1 IH-TB (0.74), IH-PB (0.75), and IH-Total (0.76) indicated adequate discrimination. An example interpretation for the AUC value for W1 IH-TB, 0.74, is that there is a 74% chance that those with W1 suicidal desire would report higher IH-TB scores than a randomly paired person with no suicidal desire in the past week.

When predictors were examined as simultaneous predictors, W1 IH-TB (SOR = 1.63, 95% Wald CI [1.24, 2.15]) and IH-PB (SOR = 1.92, 95% Wald CI [1.44, 2.56]) were significantly associated with W1 suicidal desire. The AUC value for W1 IH-TB and IH-PB simultaneously considered (0.77) indicated adequate discrimination. See Table 3 for the unstandardized model parameters of the logistic regression analyses using W1 concurrent and W2 prospective suicidal desire as the outcomes of interest (see below).

### 2.3 | Prospective Logistic Regression Analyses

Next, W1 IH-TB, IH-PB, and IH-Total scores were examined as separate predictors of W2 suicidal desire. W1 IH-TB (SOR = 2.04, 95% Wald CI [1.50, 3.16]), W1 IH-PB (SOR = 2.37, 95% Wald CI [1.70, 3.30]), and W1 IH-Total (SOR = 2.34, 95% Wald CI [1.68, 3.25]) were significantly associated with prospective suicidal desire. The AUC value for IH-TB (0.69) indicated less than adequate discrimination, and AUC values for IH-PB (0.72) and IH-Total (0.72) indicated adequate discrimination. When examined as simultaneous predictors, IH-PB (SOR = 1.98, 95% Wald CI [1.22, 3.20]) but not IH-TB (SOR = 1.26, 95% Wald CI [0.80, 1.99]) was significantly associated with prospective suicidal desire. The AUC value for IH-TB and IH-PB simultaneously considered (0.72) indicated adequate discrimination.

Finally, when the AUC values of W1 IHS scores predicting W1 or W2 suicidal desire were contrasted within each wave, only W1 IH-Total (*χ*^2^ [1, *N* = 595] = 6.90, *p* = 0.001), and W1 IH-TB and W1 IH-PB simultaneously considered (*χ*^2^ [1, *N* = 595] = 5.90, *p* = 0.015), were significantly different than W1 IH-TB in predicting concurrent suicidal desire. See Table 4 for model parameters of chi-square analyses.

### 2.4 | Clinical Utility

**FIGURE 1 |:**
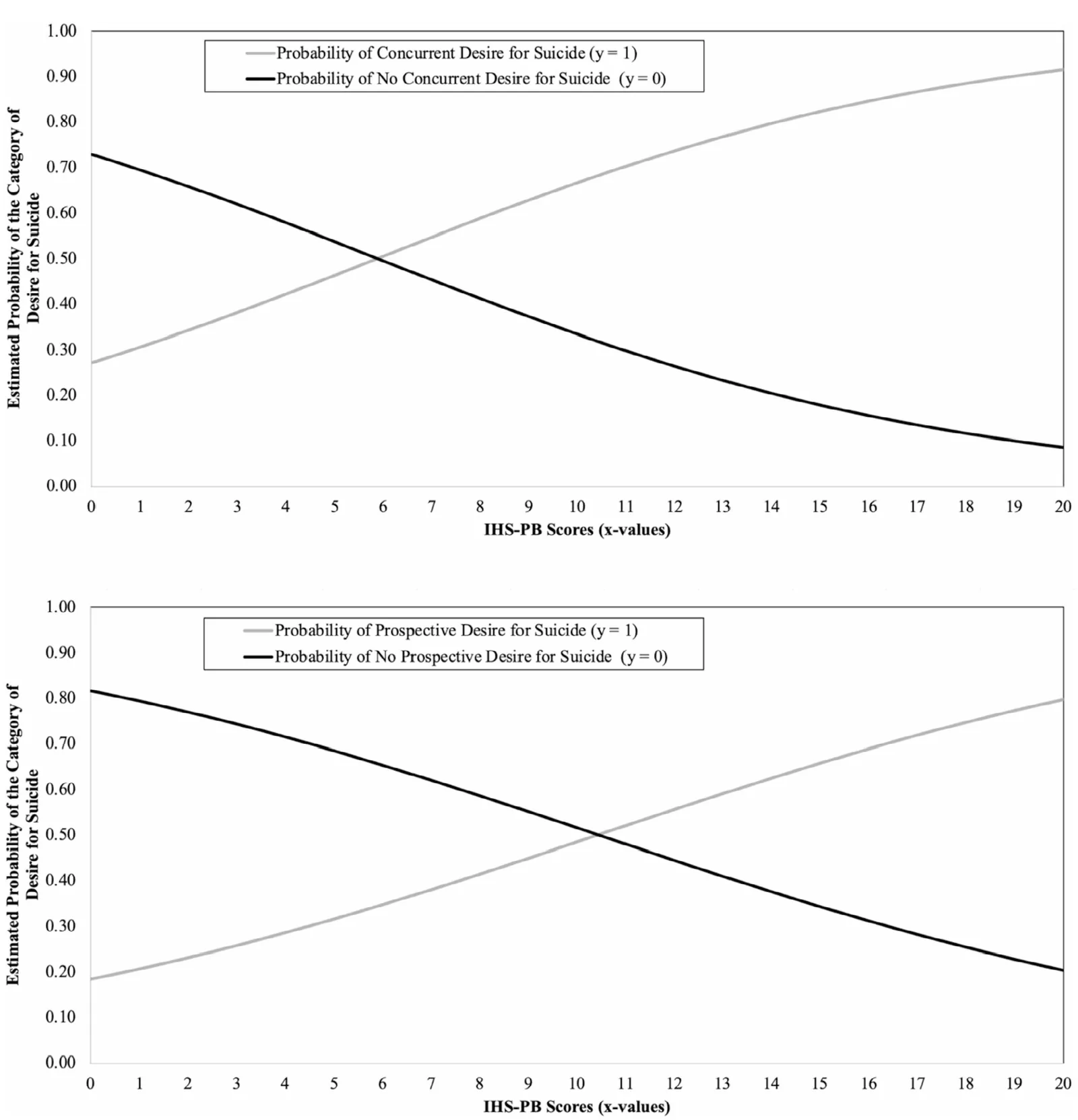
The estimated probabilities of concurrent and prospective suicidal desire across IH-TB scores. The lines in the graphs indicate the estimated probabilities of the presence or absence of desire for suicide dichotomized (0 = no desire for suicide; 1 = a weak or moderate to strong desire for suicide) across unstandardized thwarted belongingness scores concurrently considered (top graph) and prospectively considered (bottom graph); IH-TB, Interpersonal Hopelessness Scale Thwarted Belongingness Score.

**FIGURE 2 |:**
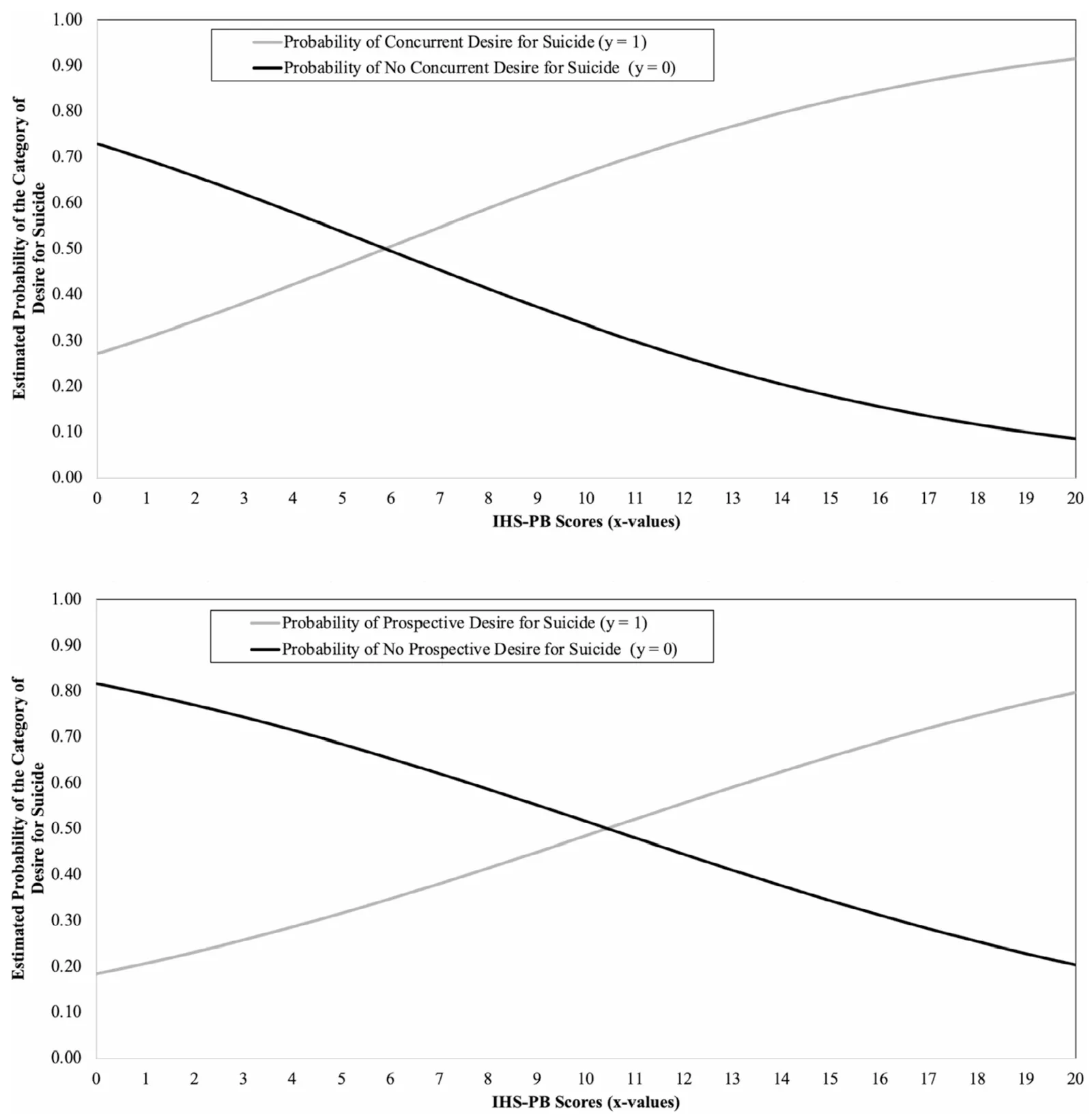
The estimated probabilities of concurrent and prospective suicidal desire across IH-PB scores. The lines in the graphs indicate the estimated probabilities of the presence or absence of desire for suicide dichotomized (0 = no desire for suicide; 1 = a weak or moderate to strong desire for suicide) across unstandardized perceived burdensomeness scores concurrently considered (top graph) and prospectively considered (bottom graph). IH-PB, Interpersonal Hopelessness Scale Perceived Burdensomeness Score.

**FIGURE 3 |:**
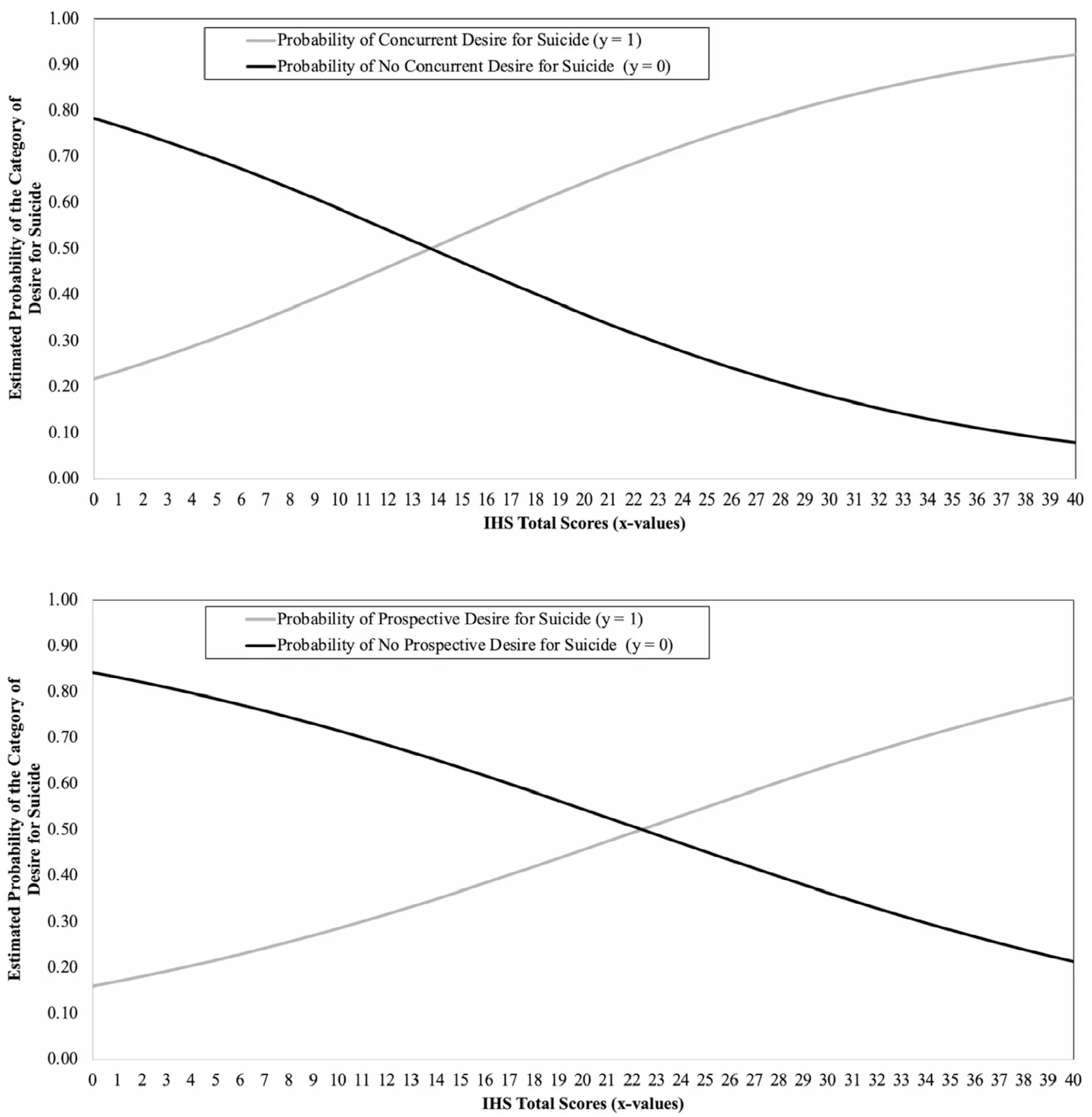
The estimated probabilities of concurrent and prospective suicidal desire across IH-Total scores. The lines in the graphs indicate the estimated probabilities of the presence or absence of desire for suicide dichotomized (0 = no desire for suicide; 1 = a weak or moderate to strong desire for suicide) across unstandardized interpersonal hopelessness total scores concurrently considered (top graph) and prospectively considered (bottom graph); IH-Total, Interpersonal Hopelessness Scale Total Score.

Clinical cutoff scores for all W1 IHS scores associated with W1 concurrent and W2 prospective suicidal desire were derived when conducting ROC curve analyses. See Table 5 for clinical cutoff scores and related indices. Estimated probabilities of concurrent and prospective suicidal desire across IH-TB, IH-PB, and IH-Total scores are provided in Figures 1–3. For example, Figure 1 (top graph) indicates that an IH-TB score of 0 (the lowest score) corresponds with a 24% chance of concurrent suicidal desire. An IH-TB score of 20 (highest score) corresponds with an 88% chance of concurrent suicidal desire. Figure 3 (bottom graph) displays graphed estimated probabilities of prospective suicidal desire across IH-Total scores for the sample. Figure 3 (bottom graph) indicates that an IH-Total score of 0 (the lowest score) corresponds with a 16% chance of suicidal desire 1 week later. An IH-Total of 39 (highest score) corresponds with a 79% chance of suicidal desire 1 week later.

### 3 | Discussion

We aimed to guide the clinical use of the IHS total and subscale scores when predicting suicidal desire concurrently (W1) and 1 week later (W2) by identifying clinical cutoff scores, developing estimated probability graphs, and comparing effect sizes of IHS scores. The IHS and its subscales demonstrated an adequate ability to predict the concurrent and prospective presence of suicidal desire (dichotomized BSS item 4). Concurrent logistic regression and AUC results indicated IHS-Total and subscales scores (i.e., IH-TB and IH-PB), as separate predictors, were significantly associated with concurrent (W1) suicidal desire. Furthermore, IH-TB, IH-PB, and IH-Total scores adequately discriminated between participants who did and did not endorse concurrent (W1) suicidal desire. When entered as simultaneous predictors, W1 IH-TB and IH-PB subscales remained significant and adequately discriminated between participants who did and did not endorse concurrent suicidal desire. Interestingly, the AUC values for IH-TB were significantly smaller than those for IH-TB and IH-PB combined, as well as the IH-Total score. This suggests that the IH-TB may provide less meaningful concurrent clinical prediction information when used in isolation than when combined with the IH-PB items, findings consistent with prior ITS studies that illustrated a similarly weaker relation between TB and SI, than that of PB to SI (Chu et al. 2017; Mitchell et al. 2020). Furthermore, concurrently, IH-TB and IH-PB, when considered simultaneously, did not perform significantly better than the IH-Total score, suggesting that either the IH-Total score or both subscales could provide the most helpful clinical information.

Consistent with the concurrent findings, prospective findings indicated that W1 IH-Total and sub-scales scores, as separate predictors, were significantly associated with the presence of suicidal desire 1 week later (W2). Additionally, W1 IH-TB, IH-PB, and IH-Total scores adequately discriminated between participants who did and did not endorse suicidal desire 1 week later (W2). When entered as simultaneous predictors, only the W1 IH-PB subscales remained significant and uniquely discriminated between participants who did and did not endorse prospective suicidal desire. None of the AUC values for the prospective analyses significantly differed, indicating a similar prediction of W2 suicidal desire based on any of the W1 IHS scores. Notably, the AUC values were smaller for the prospective analyses than the concurrent analyses, indicating lower classification accuracy and weaker effect sizes at W2 than W1, which we discuss further below in the context of clinical cutoff scores.

This study also provides important insights into the ITS. Consistent with the ITS, the IHS scores are significantly associated with suicidal desire, both concurrently and prospectively. This demonstrates that the IHS scores could be used in place of INQ-15 scores when testing hypotheses related to interpersonal hopelessness and suicidal desire that better align with the formal ITS hypothesis related to active SI. However, further research is needed to determine the optimal use of the IHS scores in statistical modeling to test the ITS hypotheses. Our findings indicate that the IH-Total score or subscale scores could be used with similar prediction strength; however, the subscale scores are significantly and strongly correlated (*r* = 0.77), which could produce multicollinearity and model interpretation difficulties (Lynam et al. 2006) if used as simultaneous predictors in the same generalized linear model. Additionally, the ITS posits that IH-PB and IH-TB are proximal risk factors for suicidal desire or active SI. This has historically been tested by including TB and PB scores from the INQ-15, for example, as mediators between other risk factors (e.g., depressive symptoms) and SI (e.g., Davidson et al. 2011; Kleiman et al. 2014). An important next step will be to conduct similar analyses using interpersonal hopelessness as a mediating variable(s) in the association between other, possibly distal risk factors and SI.

Our primary goal was to advance the clinical utility of the IHS. Broadly, our findings support the notion that the IHS can be a useful tool for evaluating risk for suicidal desire and conceptualizing possible drivers of clients’ active SI and their associated risk. These findings suggest that the IHS is best viewed as complementary to, rather than a replacement for, the INQ. Although both measures are grounded in the interpersonal theory, they assess related but distinct constructs. This distinction may be clinically meaningful, as the IHS may be more conceptually relevant to active SI. Nevertheless, future research should examine empirically whether the IHS and INQ differentially predict passive versus active SI.

To enable clinicians to interpret IHS scores more effectively, we have calculated the estimated probabilities of active SI based on various IHS scores (see Figures 1–3). This allows clinicians to easily determine the estimated probability of experiencing some level of suicidal desire based on specific raw IHS scores obtained during routine clinical assessments. Consistent with theoretical assumptions, as IHS scores increase, so does the estimated probability of endorsement of suicidal desire. We also provide clinical cutoff scores that optimally balance sensitivity and specificity, which is important to ensure accurate identification of individuals at risk of wanting to end their life while minimizing false positives that would lead to unnecessary intervention. For W1 suicidal desire, concurrent analyses indicated that W1 IH-TB has a cutoff score of 13, the IH-PB has a cutoff score of 12, and the IH-Total score has a cutoff score of 26. For W2 suicidal desire, prospective analyses indicated that both W1 IH-TB and IH-PB have a cutoff score of 9, and the IH-Total score has a cutoff score of 17. Our concurrent and 1-week prospective analyses also align with typical outpatient suicide-specific psychotherapy assessment schedules, where assessments are administered weekly and sessions are held once weekly (Jobes 2023; Rudd et al. 2015). However, it is important to consider that these cut scores are based on the sample in the current study, our outcome of interest (dichotomized BSS item 4), and the inclusion criteria (i.e., adults who endorsed past-year SI).

Notably, the cut scores for the prospective analyses are all lower than the concurrent analyses. As stated above, the AUC values were smaller for the prospective analyses than the concurrent analyses, indicating lower classification accuracy and weaker effect sizes at W2 than W1. This attenuation over time is consistent with prior research using intensive longitudinal designs, which has shown that the predictive power of suicide risk factors often decreases across time points, particularly when controlling for previous SI (e.g., Hallensleben et al. 2019; Kleiman et al. 2017). Similarly, all the Youden’s J Index values and clinical cutoff scores for the W1 IHS scores when predicting W2 presence of suicidal desire were lower than when predicting W1 suicidal desire. To some degree, AUC values, the Youden’s J Index values, and clinical cut scores are associated, such that when prediction is weaker, AUC values are smaller, and the Youden’s J Index values tend to be smaller (Yin and Vogel 2017). Additionally, the percentage of participants with some degree of suicidal desire decreased from W1 (58.8%) to W2 (44.5%); in other words, pertaining to the desire to end one’s life, the W2 sample is less clinically severe than W1. When the prevalence of a condition decreases, Youden’s J Index can also decrease because it favors specificity (true negatives) over sensitivity (true positives), which is consistent with our data and W2 sensitivity and specificity values. In other words, when the prevalence of a condition is low (or when a sample is lower risk for a given condition), clinical cutoff values will allow more false positives to avoid missing true positives because the condition is rarer and more difficult to detect (Hassanzad and Hajian-Tilaki 2024). Generally, it will be important to replicate the current study findings and clinical cutoff scores, given that the percentage of individuals who endorse some degree of suicidal desire (which can also be assessed in different ways) can vary dramatically in different settings and populations.

Importantly, the authors are not suggesting the IHS should replace other measures in a suicide risk assessment battery, nor that the IHS should be used as the sole assessment of risk for SI. Instead, we propose that the IHS be continually studied within a clinical context to further the field’s understanding of the processes involved in the development of suicidal thinking (and suicidal desire specifically) that are not otherwise covered in prior ITS literature. Clarifying the psychological factors and motivations that facilitate the transition from SI to suicidal behavior (Klonsky et al. 2018) may be particularly beneficial in improving current suicide risk assessment and screening practices that function beyond prediction, as well as for informing current treatment paradigms (e.g., treatment planning, goal setting). Given that prior studies have indicated significant correlations between IHS scores and an array of cognitive and affective risk factors for suicidal thoughts (Mitchell et al. 2024), additional research is needed that directly compares the IHS to other suicide risk assessment measures, such as other theory-driven assessments of defeat, entrapment, suicide cognitions, or thwarted interpersonal needs. However, the IHS may provide useful clinical insight based on item content and responses related to interpersonal hopelessness.

Once interpersonal hopelessness is identified, it may be a useful intervention target to reduce suicidal desire and SI intensity. Cognitive-Behavioral Therapy for suicide prevention (CBT-SP), a method that incorporates elements of traditional CBT with other evidence-based suicide prevention techniques (e.g., safety planning, chain analysis, skill building, relapse prevention; Stanley et al. 2009), has been shown to not only decrease SI but also reduce general hopelessness (Handley et al. 2013). Given that CBT-SP addresses cognitive rigidity more broadly, clinicians treating clients with SI who have high interpersonal hopelessness could consider tailoring CBT-SP toward modifying thoughts stemming from IH-TB and/or IH-PB in conjunction with other coping skills. Given the association between general hopelessness and interpersonal difficulties (see Miller et al. 2014) and the psychosocial implications of the ITS, interpersonal therapy (IPT) may be a useful treatment option for clinicians treating individuals experiencing IH-TB and/or IH-PB. This is further supported by prior evidence that IPT is similarly effective in decreasing general hopelessness and SI when compared to traditional CBT (Cuijpers et al. 2013). However, further research is needed to test if CBT-SP and IPT reduce interpersonal hopelessness and if these reductions account for subsequent reductions in SI. Investigating brief interventions that enhance or cultivate resilience against psychosocial stressors may also be a viable clinical target to reduce interpersonal hopelessness, as resiliency interventions have been shown to buffer against general hopelessness more broadly as well (Collazzoni et al. 2020; Huen et al. 2015).

### 3.1 | Limitations

This study has limitations that should be considered in future investigations of the IHS and its clinical utility. First, our findings may not generalize, as most participants were White, all were US adults, and all endorsed past-year SI. Participants were recruited online rather than from clinical settings; thus, findings should be interpreted as preliminary evidence of potential clinical utility in a higher-risk community sample. Furthermore, retention from W1 to W2 was modest (64% attrition). Although reasons for non-participation were not assessed, attrition may have been influenced by participant burden, as individuals completed both self-report measures and a suicide Stroop task at each wave, and the task required completion on a personal computer and included suicide-related (suicide, dead, funeral) and negatively valenced words (alone, rejected, stupid) as stimuli. Also, because W2 analyses were based on data from those not lost to attrition, lower suicidal desire severity at W2 may partly reflect attrition and sample composition differences. To facilitate broader clinical application, future studies examining specific clinical samples (e.g., those involved in inpatient or outpatient mental health care) will be necessary to firmly establish cutoff scores.

Second, although our findings, specifically that IH-TB and IH-PB perform worse in prospectively predicting suicidal desire when considered separately compared to simultaneously, provide initial insight into using the total score compared to subscale scores, more research is needed in this area to best understand how to use the IHS in research and clinical settings. Third, our analyses require a dichotomous criterion variable, so we dichotomized the BSS item 4, as in previous studies (Mitchell et al. 2020); the use of single items to assess SI has limitations, including imprecise language and misclassification (see Millner et al. 2015). However, Joiner et al. (2025) suggest that some single-item SI assessments can be reasonably valid when multi-item measures are unavailable. Regardless, it will be important for future researchers to consider other SI assessments when evaluating the IHS. Although providing concurrent and prospective (1 week after W1) is a strength of the current study, future research would benefit from additional longitudinal methods, such as ecological momentary assessment (EMA) or additional follow-up time points, to further examine how interpersonal hopelessness relates to aspects of SI (Gerner et al. 2023) and suicide risk outcomes (e.g., suicidal behaviors, inpatient hospitalization). Lastly, our study solely relied on self-report assessments. Future studies should examine objective measures of social disconnection (e.g., being alone vs. being with others) that may interact with perceived interpersonal hopelessness to impact SI, as has been shown with traditional interpersonal risk factors (Brown and Scott 2025; Gill et al. 2023; Glatt and Sokol 2025). Other sources of information from informants or clinical interviewers may prove useful for carefully distinguishing between types of SI and could help counter potential under-reporting of SI on self-report assessments. However, it is unlikely that under-reporting affected the results of the current study, given that participants had to endorse past-year SI to be eligible for study enrollment, and the results were anonymous and confidential.

## 4 | Conclusion

Our findings provide preliminary support for the IHS, its use in clinical settings, and evidence-based and theory-driven assessment that can inform therapeutic conceptualization. We provide clinical cutoff scores and estimated probability figures to allow for easy use by clinicians when assessing and conceptualizing a patient’s suicide risk. Additional research is needed to replicate these findings and continue bridging the divide between research and practice.

## Figures and Tables

**TABLE 1 |:** Demographic characteristics of the wave 1 only, wave 2 only, and waves 1 and 2 combined samples.

Variable	Wave 1 (*N* = 595)		Wave 2 (*N* = 215)		Waves 1 and 2 (*N* = 200)	
	*n*	%	*n*	%	*n*	%
Sex						
Male	244	41	98	45.6	89	44.5
Female	351	59	117	54.4	111	55.5
Gender						
Woman	332	55.8	111	51.6	104	52
Man	238	40	94	43.7	86	43
Transgender	7	1.2	2	0.9	2	1
Gender Non-conforming	16	2.7	7	3.3	7	3.5
Not listed	2	0.3	1	0.5	1	0.5
Race/Ethnicity						
African American/Black	51	8.6	23	10.7	20	10
White	417	70.1	142	66	138	69
American Indian Tribe or Nation	2	0.3	0	0	0	0
Asian/Asian-American	52	8.7	20	9.3	17	8.5
Latino(a)/Latinx	43	7.2	19	8.8	15	7.5
Biracial	29	4.9	10	4.7	10	5
Sexual Orientation						
Straight	443	74.5	173	80.5	160	80
Gay	18	3	3	1.4	3	1.5
Lesbian	13	2.2	5	2.3	5	2.5
Bisexual	88	14.8	23	10.7	21	10.5
Not sure	25	4.2	8	3.7	8	4
Income						
$0–$10,000	108	18.2	36	16.7	35	17.5
$10,000–$20,000	74	12.4	21	9.8	21	10.5
$20,000–$30,000	80	13.4	29	13.5	28	14
$30,000–$40,000	57	9.6	24	11.2	24	12
$40,000–$50,000	42	7.1	12	5.6	12	6
$50,000–$60,000	53	8.9	24	11.2	18	9
$60,000–$70,000	32	5.4	9	4.2	9	4.5
$70,000–$80,000	33	5.5	15	7	13	6.5
$80,000–90,000	25	4.2	10	4.7	10	5
$90,000–$100,000	26	4.4	5	2.3	4	2
Above $100,000	64	10.8	30	14	26	13
Education						
Some grade school	3	0.5	0	0	0	0
Grade school	3	0.5	0	0	0	0
Some high school	17	2.9	5	2.3	4	2
High school	115	19.3	45	20.9	44	22
Some college	168	28.2	65	30.2	61	30.5
College	194	32.6	71	33	65	32.5
Some professional school	17	2.9	2	0.9	2	1
Professional school	76	12.8	26	12.1	23	11.5
SBQ-R item 2						
Rarely (1 time)	176	29.6	55	25.6	50	25
Sometimes (2 times)	201	33.8	80	37.2	72	36
Often (3 to 4 times)	94	15.8	31	14.4	31	15.5
Very often (5 or more times)	124	20.8	49	22.8	47	23.5

*Note:* SBQ-R item 2 = Item 2 from the Suicidal Behavior Questionnaire-Revised (“How often have you thought about killing yourself in the past year?”).

Percentages may not add up to 100% due to missing data on some demographic variables.

**TABLE 2 |:** Bivariate correlations among study variables.

Variable	1	2	3	4	5
1. W1 IH-TB	—				
2. W1 IH-PB	0.77**	—			
3. W1 IH-total	0.94**	0.94**	—		
4. W1 BSS item 4	0.42**	0.43**	0.45**	—	
5. W2 BSS item 4^a^	0.33**	0.38**	0.38**	0.51**	—
W1 M	10.00	8.59	18.59	—	—
W1 SD	6.11	6.04	11.43	—	—
W1 observed range	0–20	0–20	20–40	—	—

*Note:* IH-PB, Interpersonal Hopelessness Scale Perceived Burdensomeness Score; IH-TB, Interpersonal Hopelessness Scale Thwarted Belongingness Score; IH-Total, Interpersonal Hopelessness Scale Total Score; W1 BSS Item 4, Wave 1 Beck Scale for Suicide Ideation Item 4 Dichotomized (0 = no desire for suicide; 1 = a weak or moderate to strong desire for suicide); W1, Wave 1; W2 BSS Item 4, Wave 2 Beck Scale for Suicide Ideation Item 4 Dichotomized (0 = no desire for suicide; 1 = a weak or moderate to strong desire for suicide); W2, Wave 2.

a Correlations with W2 BSS Item 4 only include participants with Wave 1 and 2 data (*N* = 200).

** *p* < 0.01.

**TABLE 3 |:** Unstandardized interpersonal hopelessness thwarted belongingness, interpersonal hopelessness perceived burdensomeness, and interpersonal hopelessness total scale scores as separate predictors of concurrent (wave 1) and prospective (wave 2) suicidal desire.

Parameter	Logit	SE	Wald *χ*^2^	*p*	OR	OR 95% CI	AUC	AIC	BIC	Intercepts
*Outcome: wave 1 BSS item 4* (*suicidal desire*)										
W1 separate predictors										
IH-TB	0.16	0.02	90.62	< 0.001	1.17	[1.13, 1.21]	0.74	701.33	707.20	−1.13
IH-PB	0.17	0.02	93.84	< 0.001	1.18	[1.14, 1.22]	0.75	693.27	699.10	−0.99
IH-Total	0.09	0.01	101.82	< 0.001	1.1	[1.09, 1.12]	0.76	681.38	687.25	−1.28
W1 simultaneous predictors										
IH-TB and IH-PB	0.08	0.02	12.06	0.001	1.08	[1.04, 1.13]	0.77	682.96	691.77	−1.27
	0.11	0.02	19.95	< 0.001	1.11	[1.06, 1.17]				
*Outcome: wave 2 BSS item 4 (suicidal desire)*										
W1 separate predictors										
IH-TB	0.12	0.03	20.12	< 0.001	1.12	[1.07, 1.18]	0.69	256.39	262.26	−1.45
IH-PB	0.14	0.03	25.76	< 0.001	1.15	[1.09, 1.22]	0.72	249.16	255.03	−1.49
IH-Total	0.07	0.02	25.52	< 0.001	1.08	[1.05, 1.11]	0.72	249.28	255.15	−1.67
W1 simultaneous predictors										
IH-TB and IH-PB	0.04	0.04	0.97	0.324	1.04	[0.96, 1.12]	0.72	250.20	259.00	−1.62
	0.11	0.04	7.76	0.005	1.12	[1.03, 1.21]				

Abbreviations: BSS Item 4, Beck Scale for Suicide Ideation Item 4 Dichotomized (0 = no desire for suicide; 1 = a weak or moderate to strong desire for suicide); IH-PB, Interpersonal Hopelessness Scale Perceived Burdensomeness Score; IH-TB, Interpersonal Hopelessness Scale Thwarted Belongingness Score; IH-Total, Interpersonal Hopelessness Scale Total Score; W1, Wave 1; W2, Wave 2.

**TABLE 4 |:** Chi-square contrast tests for interpersonal hopelessness thwarted belongingness, interpersonal hopelessness perceived burdensomeness, and interpersonal hopelessness total scale scores as separate predictors of concurrent (wave 1) and prospective (wave 2) suicidal desire.

Contrast	*χ*^2^ (1, 595)	*p*

*Outcome variable: wave 1 (w1) suicidal desire*		
W1 IH-TB vs. IH-PB	0.40	0.530
W1 IH-TB vs. IH-Total	6.90	0.001
W1 IH-PB vs. IH-Total	1.74	0.187
W1 IH-TB and IH-PB vs. IH-TB	5.90	0.015
W1 IH-TB and IH-PB vs. IH-PB	1.69	0.193
W1 IH-TB and IH-PB vs. IH-Total	< 0.01	0.309

Contrast	*χ*^2^ (1, 200)	*p*

*Outcome variable: wave 2 (w2) suicidal desire*		
W1 IH-TB vs. IH-PB	1.14	0.285
W1 IH-TB vs. IH-Total	2.90	0.089
W1 IH-PB vs. IH-Total	0.06	0.813
W1 IH-TB and IH-PB vs. IH-TB	1.91	0.167
W1 IH-TB and IH-PB vs. IH-PB	< 0.01	0.953
W1 IH-TB and IH-PB vs. IH-Total	< 0.01	0.678

Abbreviations: BSS, Beck Scale for Suicide Ideation; IH-PB, Interpersonal Hopelessness Scale Perceived Burdensomeness Score; IH-TB, Interpersonal Hopelessness Scale Thwarted Belongingness Score; IH-Total, Interpersonal Hopelessness Scale Total Score; Suicidal Desire, BSS Item 4, Beck Scale for Suicide Ideation Item 4, dichotomized (0 = no desire for suicide; 1 = a weak or moderate to strong desire for suicide); W1, Wave 1; W2, Wave 2.

**TABLE 5 |:** Receiver operating characteristic curve analyses statistics and the clinical cutoff scores of unstandardized interpersonal hopelessness thwarted belongingness and interpersonal hopelessness perceived burdensomeness scores as separate predictors of concurrent (wave 1) and prospective (wave 2) suicidal desire.

	W1 suicidal desire (BSS item 4)	W2 suicidal desire (BSS item 4)
W1 IH-TB maximum Youden J index	0.39	0.33
W1 IH-TB sensitivity	0.82	0.63
W1 IH-TB specificity	0.57	0.70
W1 IH-TB area under the curve	0.74	0.69
W1 IH-TB PPV	0.73	0.63
W1 IH-TB NPV	0.69	0.70
**W1 IH-TB cutoff score**	**13**	**9**
W1 IH-PB maximum Youden J index	0.41	0.36
W1 IH-PB sensitivity	0.69	0.62
W1 IH-PB specificity	0.72	0.74
W1 IH-PB area under the curve	0.75	0.72
W1 IH-PB PPV	0.78	0.66
W1 IH-PB NPV	0.62	0.71
**W1 IH-PB cutoff score**	**12**	**9**
W1 IH-Total maximum Youden J index	0.42	0.40
W1 IH-Total sensitivity	0.79	0.63
W1 IH-Total specificity	0.63	0.78
W1 IH-Total area under the curve	0.76	0.72
W1 IH-Total PPV	0.75	0.70
W1 IH-Total NPV	0.68	0.72
**W1 IH-Total cutoff score**	**26**	**17**

Abbreviations: BSS Item 4, Beck Scale for Suicide Ideation Item 4; IH-PB, Interpersonal Hopelessness Scale Perceived Burdensomeness Score; IH-TB, Interpersonal Hopelessness Scale Thwarted Belongingness Score; IH-Total, Interpersonal Hopelessness Scale Total Score; Dichotomized (0 = no desire for suicide; 1 = a weak or moderate to strong desire for suicide); NPV = Negative Predictive Value; PPV = Positive Predictive Value; W1 = Wave 1; W2 = Wave 2.

## Data Availability

The data that support the findings of this study are available from the corresponding author upon reasonable request.
